# Supplementary material for: Dealing with radiation risks in systemic cancer treatment: Perspectives of practitioners and patients in French hospitals
Source: PLoS One. 2025 Mar 6;20(3):e0316998. doi: 10.1371/journal.pone.0316998 (PMC11884713; doi:10.1371/journal.pone.0316998)
Supplement: Attachment — Submitted filename: comments.docx [file pone.0316998.s001.docx]

**Comments for transmission to authors:**

Solenn Thircuir and co-authors conducted a qualitative study on managing risk perception and trust in systemic radionuclide therapy (SRT) for cancer treatment, specifically with 177Lu. Their research emphasizes the importance of understanding and fostering trust throughout the treatment process. The article is well-written, and the authors effectively convey their points.

Major points:

1) The manuscript is too long with limited figures. It contains only one figure, which has many details that are not adequately explained in the figure legend. Consider adding more figures, such as those showing the demographics of systemic radionuclide therapy using 177Lu in Europe and worldwide, as well as a pipeline of the treatment process and the roles of the different people involved.

2) The sample size of interviews conducted with healthcare professionals and patients was limited to French hospitals, potentially limiting the broader applicability of the findings. The study's focus on a limited number of participants in France may not fully represent the diversity of experiences with 177Lu treatment. Consider including the country name in the title and abstract.

3) The findings may be specific to the French healthcare system and may not be generalizable to other countries. Could the authors discuss in the discussion section how other developed countries address similar issues, such as waste management and the risks associated with leakage (page 9)?

4) The trust factor highlighted by the authors is very subjective and can vary between locations and individuals. The study focused on a specific type of cancer treatment (SRT with 177Lu) and may not be generalizable to other medical interventions. Could the authors suggest a quantitative method to monitor and universalize the radiation risks associated with 177Lu in the discussion section?

Minor points:

1) Some sentences are in correction mode, please check the following:

Page 13

So,_(twice)

Page 16

_Trust; _Therefore.

2) There is unnecessary capitalization in some sentences.
   Page 5

“….thErapeutic protocols with Lu-177 for MNETs, through the development of

advanced computational tools and a portable detection sYstEm”

3) Correct the following:

Page 8

“..manipulator, + “
