# Supplementary material for: Dealing with radiation risks in systemic cancer treatment: Perspectives of practitioners and patients in French hospitals
Source: PLoS One. 2025 Mar 6;20(3):e0316998. doi: 10.1371/journal.pone.0316998 (PMC11884713; doi:10.1371/journal.pone.0316998)
Supplement: Attachment — Submitted filename: Response to Reviewers.docx [file pone.0316998.s002.docx]

Response to Reviewers

Solenn Thircuir

University of California, Santa Cruz

September 24, 2024

Dear Reviewers,

We sincerely thank you for your thorough reviews and constructive feedback on our manuscript, "Taming Risk Perception and Trust in Systemic Radionuclide Therapy for Cancer Treatment with 177Lu." We have carefully considered all your comments and made revisions accordingly. Below, we address each of your points in detail.

**Response to Reviewer 1**

1. **Technical soundness and data support**:

We acknowledge your concern regarding the manuscript's length relative to the limited number of figures. To improve clarity and understanding, we have added additional figures illustrating the demographics of systemic radionuclide therapy using 177Lu in Europe, along with a table highlighting the roles of various stakeholders involved in the treatment process in the methodology section.

2. **Sample size and generalizability**:

We appreciate your observation about the survey’s focus on French hospitals. To enhance the manuscript’s applicability, we have specified the country name in both the title and abstract. Additionally, we have included a discussion on the potential limitations regarding generalizability and how other Western countries address similar issues, which is now reflected in the revised discussion section.

3. **Subjectivity of trust**:

In the revised discussion, we have suggested that a quantitative method for monitoring and universalizing the radiation risks associated with 177Lu would further our analysis. This addition aims to foster dialogue and provide a more robust framework for understanding trust dynamics across different medical interventions.

4. **Minor points**:

In response to your suggestions for minor language editing, we conducted a thorough review of the entire manuscript, correcting misspellings and typos, removing redundant sentences, and ensuring proper capitalization throughout.

**Response to Reviewer 2**

1. **Title and abstract**:

We have removed “Taming” Risk? from the title as per your suggestion and replaced it with “Dealing with Radiation Risks in Systemic Cancer Treatment: Perspectives of Practitioners and Patients.” The abstract has been rewritten to include key results and findings from our study, providing a clearer overview of our work and its implications.

2. **Language and editing**:

To address your concerns about the language, we performed a thorough review of the entire text and made significant edits for clarity. We also engaged a professional language editor to ensure that the manuscript meets high linguistic standards.

3. **Data availability**:

We would like to clarify that while we aim to adhere to data availability requirements, qualitative data cannot be made fully available due to the sensitivity of personal and health information involved.

4. **Introduction reorganization**:

The introduction has been reorganized to place the aim of the study at the end, following the historical context. This restructuring improves the flow of information and provides a clearer focus for readers.

In sum, we have thoroughly engaged your suggestions.  Thank you for considering our revised article re-submission. We believe that these revisions comprehensively address the reviewers’ concerns and enhance the overall quality of the manuscript. Thank you once again for your valuable feedback. We look forward to your further consideration of our revised submission.

Sincerely,

Solenn Thircuir, Héloïse Pillayre, Johannes Starkbaum, and Erich Griessler
