# Supplementary material for: Dealing with radiation risks in systemic cancer treatment: Perspectives of practitioners and patients in French hospitals
Source: PLoS One. 2025 Mar 6;20(3):e0316998. doi: 10.1371/journal.pone.0316998 (PMC11884713; doi:10.1371/journal.pone.0316998)
Supplement: Attachment — Submitted filename: Comments.docx [file pone.0316998.s003.docx]

The authors have made significant changes to the manuscript after the first round of review, which is highly appreciated. However, here are my comments after reviewing the manuscript for the second time.

1) Authors should address each reviewer comment individually, rather than providing general responses about the changes made.

For example:

Reviewer Comment 1:

Author's Response:

2) Replace 'graph 1' with 'figure 1' and add a legend for figure 1. Additionally, rename the current figure 1 to figure 2.
3) The previous comment regarding the figure 1 legend has not been addressed. The current diagram of the brachytherapy department is too complex. Please either elaborate on the figure 1 legend, even if the information is also provided in the results, or simplify figure 1 itself. Ensure the format follows conventional standards.
   For example:
   **Figure 1: Zones of a brachytherapy department**. Add detailed description here.
4) Please review the title to ensure it includes any necessary punctuation marks that might be missing.
5) Replace “pathway” with “pipeline”.
6) Double-check punctuation throughout the manuscript, particularly in newly modified text.
