# Supplementary material for: Dealing with radiation risks in systemic cancer treatment: Perspectives of practitioners and patients in French hospitals
Source: PLoS One. 2025 Mar 6;20(3):e0316998. doi: 10.1371/journal.pone.0316998 (PMC11884713; doi:10.1371/journal.pone.0316998)
Supplement: Attachment — Submitted filename: Response to Reviewer 2.docx [file pone.0316998.s004.docx]

Response to Reviewer 2

Solenn Thircuir

University of California, Santa Cruz

November 15, 2024

Dear Reviewer,

We sincerely thank you for your thoughtful feedback and for accepting the revised version of our manuscript, *"*Dealing with Radiation Risks in Systemic Cancer Treatment: Perspectives of Practitioners and Patients in French Hospitals."

We deeply appreciate your acknowledgment that the revisions have adequately addressed your comments from the previous review round. Your constructive suggestions have been invaluable in refining our work, and we are delighted that the manuscript is now ready for publication.

Thank you once again for your time, expertise, and support throughout the review process.

Sincerely,

Solenn Thircuir, Héloïse Pillayre, Johannes Starkbaum, and Erich Griessler
